# The Impact of Temperature and Humidity on the Performance and Physiology of Laying Hens

**DOI:** 10.3390/ani11010056

**Published:** 2020-12-30

**Authors:** Da-Hye Kim, Yoo-Kyung Lee, Sang-Ho Kim, Kyung-Woo Lee

**Affiliations:** 1 Department of Animal Science and Technology, Konkuk University, 120 Neungdong-ro, Gwangjin-gu, Seoul 05029, Korea; kdh142536@naver.com; 2 Rural Development of Administration (NIAS-RDA), National Institute of Animal Science, Iseo-myeon, Wanju-gun, Jeollabuk-do 55365, Korea; yoo3930@korea.kr (Y.-K.L.); kims2051@korea.kr (S.-H.K.)

**Keywords:** heat stress, temperature-humidity index, laying performance, egg quality, laying hens, stress indicators

## Abstract

**Simple Summary:**

The present study investigated whether the temperature-humidity index (THI) influences the production parameters and physiology of laying hens. Two environmental conditions combining high temperature with low relative humidity (T_L_H_H_75) or vice versa (T_H_H_L_75), with the same THI value (75), were considered. The same THI value indicated equal thermal stress for laying hens. Neither T_L_H_H_75 nor T_H_H_L_75 affected laying performance, including egg production, egg weight, and egg mass, feed intake, feed conversion ratio, plasma biochemical parameters, and stress indicators. Our study suggests that laying hens exposed to the same THI values will receive similar thermal stresses. The results of this study will serve as a scientific basis for management decisions and handling laying hens under thermally challenging conditions.

**Abstract:**

We investigated the effect of different ambient temperatures and relative humidity (RH) with the same temperature-humidity indices (THI) on laying performance, egg quality, heterophil to lymphocyte ratio (H/L ratio), corticosterone (CORT) concentration in blood, yolk, and albumen, and plasma biochemical parameters of laying hens. Commercial hens (Hy-Line Brown; *n* = 120), aged 60 weeks, were allocated to two environmental chambers. Laying hens were subjected to either one of two thermal treatments—26 °C and 70% RH (T_L_H_H_75) or 30 °C and 30% RH (T_H_H_L_75) for 28 days—with the same THI of 75. Neither T_L_H_H_75 nor T_H_H_L_75 affected laying performance, including egg production, egg weight, egg mass, feed intake, and feed conversion ratio (*p* > 0.05). Plasma biochemical parameters such as total cholesterol, high-density lipoprotein cholesterol, triglycerides, calcium, magnesium, and phosphorus were not altered by the environmental treatments (*p* > 0.05). As for stress indicators, both environmental regimes failed to affect blood H/L ratio and CORT levels in plasma, yolk, and albumen (*p* > 0.05), although albumen CORT levels were elevated (*p* < 0.05) in T_L_H_H_75 group at day 7. Hence, our study suggests that laying hens performed and responded similarly when exposed to either T_L_H_H_75 or T_H_H_L_75 characterized by the same THI. These results can serve as a scientific basis for management decisions and handling laying hens under thermally challenging conditions.

## 1. Introduction

The optimum temperature (thermoneutral zone) for laying hens allowing optimal performance is between 19 and 22 °C [1]. Laying hens exposed to ambient temperatures above the thermoneutral zone experience heat stress that triggers physiological defense mechanisms, including panting [2]. Moreover, laying hens are vulnerable to heat stress due to their extended production cycle of up to 74 weeks of age [2], lack of sweat glands, feathers, and generation of high metabolic body heat [3]. In addition, heat stress per se affects egg production and quality [4,5], as well as physiological [6,7] and stress responses [8] of laying hens. When subjected to heat stress, laying hens significantly reduce their feed intake (38.8%), laying performance (5.0%), and body weight (5.2%) at 24 weeks of age [4]. Reduction in egg quality and production can be primarily mediated by a decrease in available calcium, as heat stress decreases calcium intake and absorption at the gut level [9]. Finally, exposure of chickens to heat stress increases the secretion of corticosterone (CORT), a major stress hormone in chickens, via activation of the hypothalamic-pituitary-adrenal (HPA) axis [10].

The temperature-humidity index (THI), a heat or stress index, has been used to assess the effect of environmental conditions on animals’ thermoregulatory mechanisms and to prevent temperature stress [11,12]. Both ambient temperature and relative humidity (RH) are used as variables to calculate THI values [11,12,13], where temperature is considered to contribute more to THI values than humidity. Zulovich and DeShazer [11] developed a THI chart for laying hens based on egg production levels and physiological responses. The THI chart classifies stress into four levels: comfort (THI < 70), alert (THI 70–75), danger (THI 76–81), and emergency (THI > 81) zones. This chart can be used to assess the stress levels of laying hens subjected to each THI zone. Indeed, Kang et al. [14] recently reported on the negative effects of increasing THI conditions on the mortality and panting rate in laying hens and concluded that the THI chart can be used to assess the effect of heat stress in laying hens within the poultry house.

Considering the lack of sweat glands and heavy reliance on panting for thermoregulation in chickens, the THI can be used to assess the performance and physiological response of laying hens. Theoretically, the same THI values calculated with different combinations of temperature and RH expose the laying hens to the same thermal (i.e., heat) stress. Furthermore, environmental RH is a triggering factor to heat stress [15]. However, it is considered ironic that humidity per se is often neglected in designing heat stress environments, as it is difficult to control the RH in an experimental setting. Thus, we hypothesized that the same THI values with different combinations of ambient temperature and RH would equally affect laying hens with regard to laying performance, egg quality, and stress indicators (i.e., heterophil to lymphocyte (H/L) ratio and CORT). To address this hypothesis, we investigated the time-dependent changes in productive and physiological responses throughout heat stress, which seemed to be a robust approach to evaluate the stress response, if any, of laying hens exposed to the same THI. However, it may be beyond the scope of this study to determine whether the designed THI conditions are naturally present within the poultry houses, which are affected by multiple factors, including weather, laying hens housing, fecal droppings, water spray, and ventilation.

## 2. Materials and Methods

### 2.1. Birds and Experimental Design 

One hundred and twenty 60-week-old laying hens (Hy-Line Brown) were housed in two environmental chambers. Each chamber was equipped with a heater, an air-conditioner, a humidifier, a dehumidifier, a main controller, and 20 one-tier cages, 1 m above the floor. Each cage had dimensions of 41 cm × 37 cm × 40 cm (length × depth × height), two nipple waterers, and a trough feeder with three hens housed per cage. Two cages were considered a replicate. Hens were initially adapted to the chambers for 2 weeks at an ambient temperature of 24 °C, RH of 50% (THI 70), and a lighting program of 16L/8D. After adaptation, one chamber was set to 26 °C with 70% RH (T_L_H_H_75), while the other was set to 30 °C with 30% RH (T_H_H_L_75) for 28 days. Both thermal treatments had the same THI of 75. The THI was calculated using the formula of Zulovich and DeShazer [8]; THI = 0.6 T_db_ + 0.4 T_wb_, where THI = temperature-humidity index in °F, T_db_ = dry-bulb temperature in °F, and T_wb_ = wet-bulb temperature in °F. Temperature and humidity loggers (MHT-381SD; Lutron Electronic Enterprise Co., Taipei, Taiwan) were placed in each chamber. Corn-soybean meal-based commercial layer diets were used (Table 1). Laying hens had ad libitum access to water and feed throughout the experimental period. The body weights of the laying hens were measured at the beginning and end of the experiment.

### 2.2. Productive Performance and Egg Quality

Eggs were collected daily at 09:00 h and weighed per replicate. Hen-day egg production was calculated as the total eggs laid/hen-days multiplied by 100. The feed conversion ratio was expressed as kg of feed consumed per kg of eggs produced and kg of feed consumed per 12 eggs laid. Eggs (*n* = 6/replicate) were collected on three consecutive days at the beginning of the experiment and then weekly to measure egg quality. The egg specific gravity was determined using the saline flotation method [16] with six salt solutions (1.100, 1.090, 1.080, 1.070, 1.060, and 1.050) at room temperature. The Haugh unit, eggshell strength, eggshell thickness, and yolk color were determined using a digital egg tester (DET-6000, Navel, Kyoto, Japan). The eggshells were cleaned of any adhering albumen using absorbent paper and dried at room temperature to determine eggshell weight.

### 2.3. Measurement of Blood Parameters

At days 0, 2, 3, 7, 8, 12, 14, 16, 21, and 28, ten birds per treatment were randomly selected for blood collection. Blood was drawn from the wing vein into a heparinized tube, and special care was taken not to sample the same hens within 3 weeks. One drop of whole blood was smeared on a glass slide and dyed using a Differential Quik Stain Kit (Polysciences Asia-Pacific, Inc., Taipei, Taiwan), and heterophils and lymphocytes (for H/L ratio) were counted under a light microscope (Olympus BX 43, Olympus Optical Co. Ltd., Tokyo, Japan). Plasma was separated by centrifugation at 200 × *g* for 15 min and stored at −20 °C until analysis. Plasma CORT concentrations were determined using a CORT ELISA kit (Enzo life science Inc, ADI-901-097, Farmingdale, NY, USA) according to the manufacturer’s instructions. In addition, plasma samples collected on days 0, 3, 7, 14, 21, and 28 were analyzed for total cholesterol, triglycerides, high-density lipoprotein cholesterol, calcium, magnesium, and phosphorus using an automatic blood chemical analyzer (Film DRI CHEM 7000i, Fuji film, Tokyo, Japan). Nitric oxide in plasma samples was measured using a modified Griess reagent (Sigma-Aldrich, St. Louis, MO, USA) as previously described [17].

### 2.4. Corticosterone in Egg Yolk and Albumen

Three eggs per replicate were collected for CORT determination in egg yolks and albumens on days 0, 2, 3, 7, 8, 12, 14, 16, 21, and 28 following temperature treatment. The eggs were cracked open, and the yolk and albumen were separated and pooled. The pooled yolk and albumen per replicate were homogenized, and CORT concentration was measured using a CORT ELISA kit (Enzo Life Science Inc., ADI-901-097, Farmingdale, NY, USA) as described elsewhere [18,19].

### 2.5. Statistical Analysis

Two adjacent cages were considered an experimental unit. In the present study, no age effect was considered. However, it was confirmed that there were no replicate variations affecting the measured parameters. Results were presented as least square means and standard deviation. All data were analyzed using the paired t-test procedure of SAS (SAS Institute Inc., Cary, NC, USA). Correlation coefficients were estimated between stress indicators (i.e., CORT in egg yolk and plasma, and blood H/L ratio) using the correlation (CORR) procedure of SAS (SAS Institute Inc., Cary, NC, USA). Differences were considered significant at *p* < 0.05.

## 3. Results

### 3.1. Productive Performance and Egg Quality

**Figure 1:**
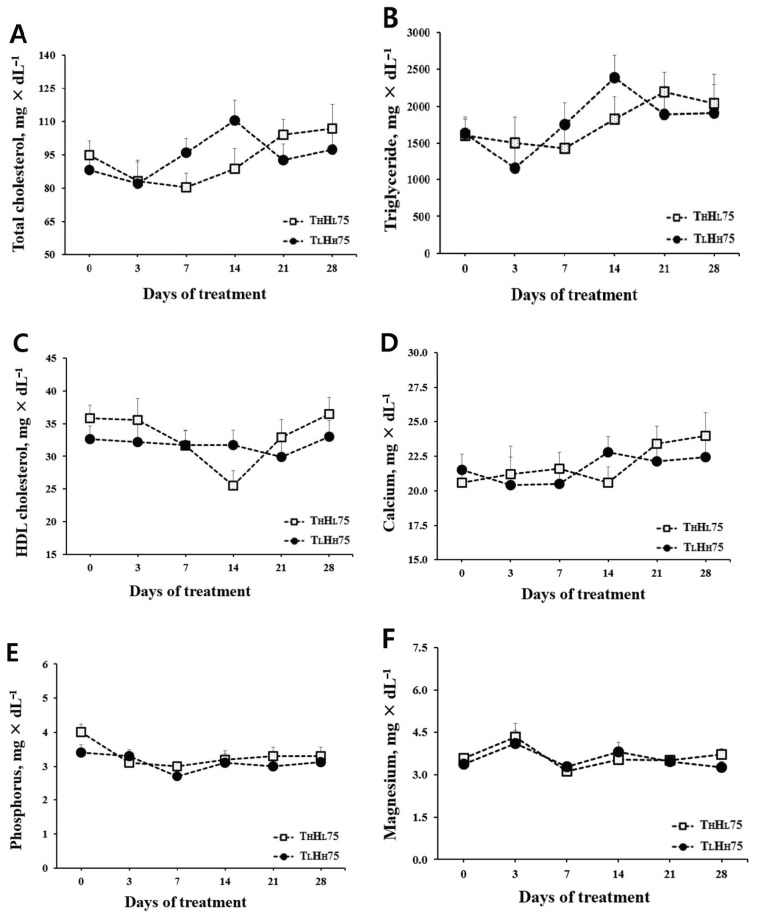

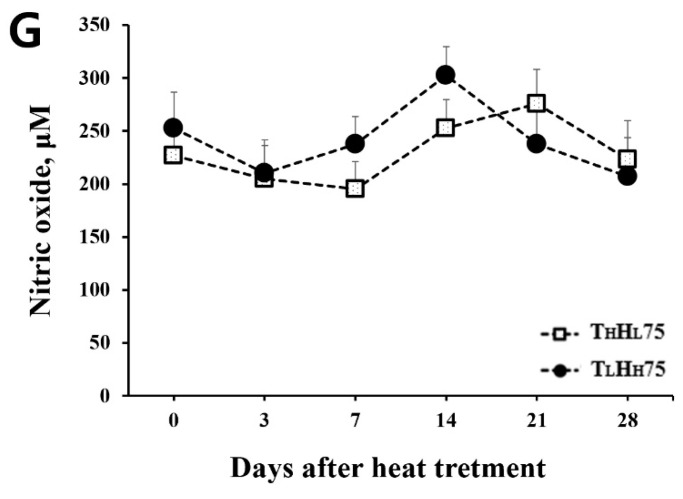
Effect of different ambient temperatures and humidity (same Temperature-Humidity Index) on plasma biochemical parameters. Laying hens were exposed to two ambient temperature conditions (26 °C, 70%; 30 °C, 30%). Error bars indicate the standard deviation of the mean (*n* = 10). T_L_H_H_75 = temperature 26 °C; relative humidity 70%; T_H_H_L_75 = temperature 30 °C; relative humidity 30%. (**A**) Total cholesterol (mg × dL^−1^). (**B**) Triglycerides (mg × dL^−1^). (**C**) High-density lipoprotein (HDL) cholesterol (mg × dL^−1^). (**D**) Calcium (mg × dL^−1^). (**E**) Phosphorus (mg × dL^−1^). (**F**) Magnesium (mg × dL^−1^). (**G**) Nitric oxide (μM).

Body weight at the beginning and end of the experiment ranged from 1.94 to 1.97 kg. Laying performance was not altered on a weekly basis between treatments and was thus presented for the entire period (Table 2). When hens were exposed to THI 75 conditions with either T_L_H_H_75 or T_H_H_L_75, no differences were observed in any of the measured variables, including hen-day egg production, egg weight, egg mass, and feed conversion ratio. Neither T_L_H_H_75 nor T_H_H_L_75 affected weekly egg quality, including the Haugh unit, eggshell strength, eggshell thickness, specific gravity, and absolute and relative eggshell weight (Figure 1).

### 3.2. Blood Chemical Parameters

**Figure 2:**
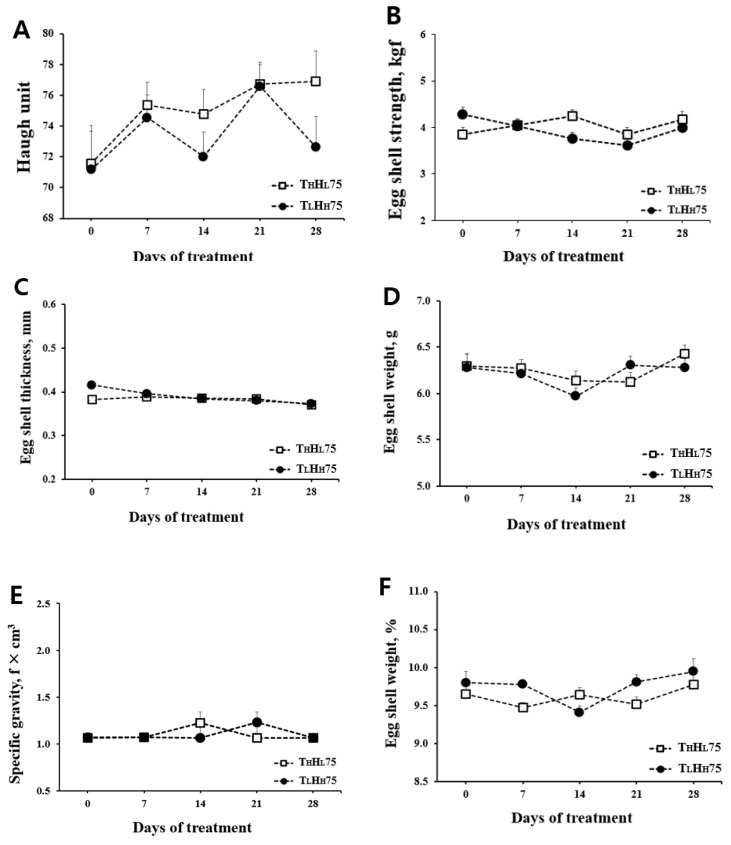
Effect of different ambient temperatures and humidity (same temperature-humidity Index) on egg quality. Laying hens were exposed to two ambient temperature conditions (26 °C, 70%; 30 °C, 30%). Error bars indicate the standard deviation of the mean (*n* = 10). T_L_H_H_75 = temperature 26 °C; relative humidity 70%; T_H_H_L_75 = temperature 30 °C; relative humidity 30%. (**A**) Haugh unit. (**B**) Eggshell strength (kgf). (**C**) Eggshell thickness (mm). (**D**) Eggshell weight (g). (**E**) Specific gravity (f × cm^3^). (**F**) Eggshell weight (%).

Neither T_L_H_H_75 nor T_H_H_L_75 affected plasma biochemical profiles, including total cholesterol, high-density lipoprotein cholesterol, triglycerides, calcium, phosphorus, and magnesium (Figure 2). Nitric oxide, an innate immunity indicator, was not altered by temperature/humidity regimes (i.e., T_L_H_H_75 or T_H_H_L_75).

### 3.3. Blood Levels of Corticosterone and Heterophil to Lymphocyte Ratio

**Figure 3:**
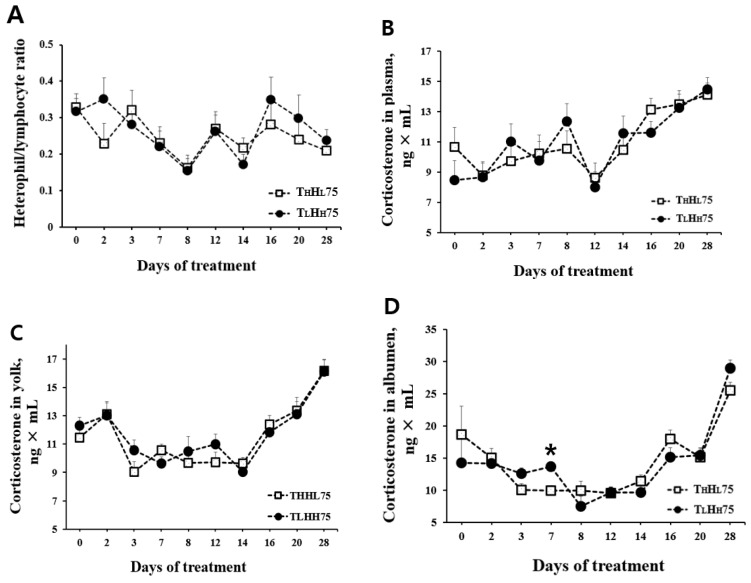
Effect of different ambient temperatures and humidity (same temperature-humidity index) on heterophil/lymphocyte ratio in blood, and corticosterone in plasma, yolk, and in albumen. T_L_H_H_75 = temperature 26 °C; relative humidity 70%; T_H_H_L_75 = temperature 30 °C; relative humidity 30%. The asterisk indicates a significant difference between the means. Error bars indicate the standard deviation of the mean (*n* = 10). (**A**) Heterophil/lymphocyte ratio. (**B**) Corticosterone in plasma (ng × mL). (**C**) Corticosterone in yolk (ng × mL). (**D**) Corticosterone in albumen (ng × mL).

Multiple stress indicators were measured to assess the stress response of laying hens exposed to either T_L_H_H_75 or T_H_H_L_75. As the response of laying hens to ambient temperature might be altered or varied during heat exposure, we assayed the H/L ratio in the blood and CORT in plasma and eggs at several time points following heat treatment. As shown in Figure 3, H/L ratio ranged from 0.155 to 0.351 for hens exposed to T_L_H_H_75 and from 0.163 to 0.329 for those exposed to T_H_H_L_75 (*p* > 0.05). Furthermore, plasma CORT levels were not altered (*p* > 0.05) in laying hens between the treatments. Plasma CORT in T_L_H_H_75-exposed hens fell within 8.01–14.47 ng × mL and those on the T_H_H_L_75 regime were 8.62–14.12 ng CORT × mL. 

### 3.4. Corticosterone in Egg Yolk and Albumen

**Figure 4:**
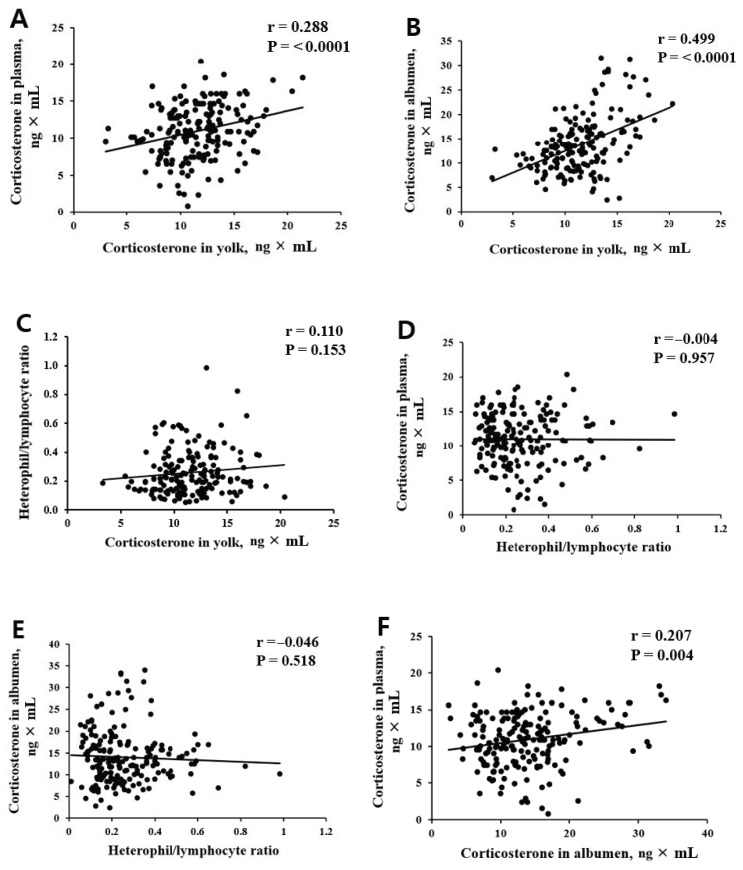
Correlation between (**A**) corticosterone in yolk and corticosterone in plasma, (**B**) corticosterone in yolk and corticosterone in albumen, (**C**) corticosterone in yolk and heterophil to lymphocyte ratio, (**D**) heterophil to lymphocyte ratio and corticosterone in plasma, (**E**) heterophil to lymphocyte ratio and corticosterone in albumen, and (**F**) corticosterone in albumen and corticosterone in plasma.

CORT can be deposited in both yolk and albumen during egg formation, which can be used to assess the stress status of laying hens [20]. The CORT concentration in the yolk and albumen are shown in Figure 3. The two THI-75 regimes (T_L_H_H_75 and T_H_H_L_75) did not affect the CORT levels deposited in the yolk. CORT levels ranged over 9.03–16.13 ng g^−1^ wet-yolk for the T_L_H_H_75-conditioned regime and 9.05–16.18 ng g^−1^ wet-yolk for the T_H_H_L_75-conditioned regime. Both temperature/RH conditions did not affect albumen CORT levels, except for albumen collected on day 7 (*p* = 0.003; Figure 3). Correlation analysis revealed a small to moderate association between plasma CORT and yolk CORT (r = 0.288, Figure 4A), yolk CORT and albumen CORT (r = 0.499, Figure 4B), and CORT in plasma and albumen (r = 0.207, Figure 4F). Negligible associations between the H/L ratio and CORT in plasma, yolk, or albumen were noted (Figure 4C–E).

## 4. Discussion

It is clear from this study that the same THI conditions created using different combinations of temperature and RH (i.e., T_H_H_L_75 or T_L_H_H_75) showed no differences in laying performance, egg quality, and serum biochemical profiles during the 28-day experiment. Except for albumen CORT at day 7, all stress indicators including H/L ratio, CORT in plasma, yolk, and albumen were not altered by the THI regimes. Thus, our results indicate that laying hens exhibit similar productive and physiological responses to different environmental conditions (i.e., T_H_H_L_75 and T_L_H_H_75) with the same THI (i.e., 75). Furthermore, our study confirms the reliability of the THI chart with the thermal (heat) indices developed for livestock and laying hens [11,21], as laying hens will theoretically receive the same intensity of stress under the same THI conditions (determined using different temperatures and RH).

It can be argued that the lack of responses to the different ambient temperatures observed in this study might be attributed to the difference in environmental temperature being 4 °C (26 °C vs. 30 °C). Indeed, Yahav et al. [22] reported that temperature, but not RH, is the most crucial factor in heat stress affecting the performance and physiology of laying hens. However, an earlier study reported that laying hens housed at 30 °C had a poor performance and exhibited altered nutrient digestibility [23] compared to hens housed at 24 °C. Similarly, we observed a negative effect of heat stress on laying hens when they were exposed to 32 °C vs. 27 °C [24]. Thus, the selected response indicators (i.e., performance, egg quality, serum parameters, and stress indicators) could have been altered if temperature, but not RH, played an essential role in influencing the performance, behavior, or physiology of laying hens in this study.

High environmental temperatures impair egg production and eggshell quality, leading to considerable economic losses in the global egg industry [25,26,27]. It has been established that laying hens exposed to heat stress reduce feed intake to minimize heat production and change blood flow from the organs to the body surface area to dissipate sensible heat [4]. In addition, heat stress impairs ovarian function by lowering ovarian weight and the number of large follicles [7,24,28] as well as plasma concentrations of minerals, including calcium, magnesium, and phosphorus [24]. As observed in this study, none of the environmental treatments affected blood biochemical parameters, indicating that neither environmental regime affected heat stress’s physiological indicators in laying hens. Thus, it is of utmost importance to monitor thermal environments (e.g., temperature and RH) within poultry houses, and the THI chart can be employed to predict heat stress in laying hens.

Heat stress increases the H/L ratio due to a low lymphocyte count and high heterophil count [15] caused by an elevated circulating CORT concentration, leading to the redistribution of lymphocytes between blood and lymphoid and non-lymphoid tissues [8]. Heat stress-exposed chickens trigger HPA axis activation, which increases blood CORT concentrations [29]. In this sense, CORT has been used as a standard indicator to assess the effect of heat stress in poultry [6,30]. CORT is the dominant glucocorticoid present in the blood, and CORT concentrations in eggs are positively correlated with maternal CORT circulation [31,32]. CORT is chronically deposited in eggs through incorporation into the yolk during the rapid yolk deposition phase of follicular development and in an acute manner via passage into the albumen at the magnum after ovulation [19,33]. In this study, except for the elevated CORT levels in albumen at day 7 following heat treatment, the H/L ratio and CORT levels in plasma, yolk, and albumen were not altered by the ambient environment during the heat exposure period. At this stage, it is not clear how albumen CORT levels were significantly increased in laying hens in the T_L_H_H_75 treatment compared with the T_H_H_L_75 treatment on day 7 following heat treatment. This finding contradicts an earlier report [22] emphasizing temperature as a dominant factor in the laying hens’ stress response. However, the fact that no consistent effect of T_L_H_H_75 vs. T_H_H_L_75 on albumen CORT was noted during the 28-d heat treatment needs to be cautiously interpreted. In addition, a moderate correlation was found between yolk CORT and albumen CORT (r = 0.499), and a low correlation was found between plasma CORT and yolk CORT (r = 0.288) and between plasma CORT and albumen CORT (r = 0.207). Our study suggests that both temperature- and RH conditions had similar effects on stress response, if any, in laying hens.

## 5. Conclusions

It can be concluded from this study that laying hens exposed to different environmental conditions with same THI (i.e., T_L_H_H_75 or T_H_H_L_75) exhibit no differences in laying performance, egg quality, and plasma biochemical profiles. As for the stress indicators, both environmental regimes did not affect blood H/L ratio and CORT levels in plasma, yolk, and albumen, although albumen CORT was elevated in T_L_H_H_75 group on day 7. Collectively, laying hens exposed to environmental conditions with the same THI values will receive similar thermal stress. Our results could help establish technical guidelines for controlling ambient temperature and humidity using information and communication technology (ICT)-based smart farms for poultry.

## Figures and Tables

**Table 1:** Ingredient and chemical composition of the basal diet.

Ingredients	%
Corn	43.0
Dried distiller grains with solubles	17.0
Wheat	5.58
Soybean meal, 45% crude protein	5.14
Corn germ meal	5.52
Rice dehulled	4.0
Rapeseed meal	3.0
Rice bran	2.0
Liquid condensed molasses solubles	1.0
Limestone	10.7
Monodicalcium phosphate	0.66
Salt	0.22
Carrier (corn)	1.25
Methionine-100%	0.06
Lysine sulfate-54%	0.25
Tryptophane-10%	0.30
Liquid choline	0.06
Vitamin mix ^1^	0.12
Mineral mix ^2^	0.14
Total	100.00
Calculated or analyzed chemical composition	
Nitrogen-corrected apparent metabolizable energy ^3^, kcal × kg	2600
Dry matter ^4^	89.2
Crude protein^4^	14.8
Calcium ^4^	5.15
Total phosphorus ^4^	0.60
Available phosphorus ^3^	0.28
Salt ^3^	0.15
Lysine ^3^	0.64
Methionine ^3^	0.32
Threonine ^3^	0.52
Tryptophan ^3^	0.16

^1^ Vitamin mixture provided following nutrients per kg of diet: vitamin A, 15,400 IU; vitamin D_3_, 3080 IU; vitamin E, 14 mg; vitamin K_3_, 1.4 mg; vitamin B_1_, 1.12 mg; vitamin B_2_, 2.8 mg; vitamin B_6_, 3.92 mg; vitamin B_12_, 0.014 mg; niacin, 56 mg; pantothenic acid, 5.6 mg; folic acid, 0.28 mg. biotin, 0.14 mg; choline, 260.4 mg. ^2^ Mineral mixture provided the following nutrients per kg of diet: Mn, 70 mg; Zn, 50 mg; Fe, 50 mg; Cu, 7 mg; I, 0.75 mg; Co, 0.4 mg; Se, 0.17 mg. ^3^ Calculated values. ^4^ Analyzed values.

**Table 2:** Effect of different ambient temperatures with the same temperature-humidity index (THI = 75) on laying performance of laying hens ^1.^

Items	Thermal Treatment				*p*-Value
	T_L_H_H_75		T_H_H_L_75		
	Mean	SD	Mean	SD	
0 to 28 days					
Hen-day egg production, %	75.73	9.70	74.82	11.39	0.849
Egg weight, g/egg	66.97	3.96	65.43	2.26	0.299
Egg mass, g/day	50.76	7.26	49.00	7.97	0.612
Feed intake, g/day/bird	115.8	5.51	117.4	7.64	0.591
FCR, kg/kg	2.32	0.34	2.44	0.31	0.427
FCR, kg/12 eggs	1.86	0.23	1.91	0.23	0.605

^1^*n* = 10 replicates per treatment. T_L_H_H_75 = temperature 26 °C; relative humidity 70%; T_H_H_L_75 = temperature 30 °C; relative humidity 30%. FCR = feed conversion ratio. SD = standard deviation.

## Data Availability

Data is contained within the article.
